# Extensive Supratentorial Hemorrhages Following Posterior Fossa Meningioma Surgery

**DOI:** 10.4103/2006-8808.73624

**Published:** 2010

**Authors:** Amit Agrawal, Anand Kakani, Kaushik Ray

**Affiliations:** *Department of Neurosurgery, Datta Meghe Institute of Medical Sciences, Sawangi (Meghe), Wardha, Maharashtra, India*; 1 *Department of Surgery, Datta Meghe Institute of Medical Sciences, Sawangi (Meghe), Wardha, Maharashtra, India*

**Keywords:** Meningioma, posterior fossa surgery, supratentorial hemorrhage

## Abstract

Remote supratentorial hematoma soon after posterior fossa surgery for the removal of a space-occupying lesion is a rare but dramatic and dreaded complication, carrying significant morbidity and mortality. A 47-year-old woman presented with headache of 1-year duration that worsened over last 2 months, progressive ataxia of 2 months’ duration, blurring and diminution of vision of 2 months’ duration and forgetfulness of 2 months’ duration. Fundus showed bilateral papille dema, and visual acuity was 6/9 in both eyes. She had left-sided cerebellar signs. There were no focal motor or sensory neurological deficits. MRI brain with contrast showed a large posterior fossa tumor with obstructive hydrocephalus. The patient underwent left paramedian suboccipital craniectomy in prone position with left side up. In the immediate postoperative period, the patient did no recover from anesthesia and was persistently drowsy. Immediate repeat CT scan showed diffuse subarachnoid hemorrhage spread all over the bilateral cerebral hemispheres with diffuse cerebral edema. The patient recovered with conservative management without deficits. This case stresses the importance of early postoperative CT scan and optimal management of the hemorrhage for good outcome.

## INTRODUCTION

Remote supratentorial hematoma soon after posterior fossa surgery for the removal of a space-occupying lesion is a rare but dramatic and dreaded complication, carrying significant morbidity and mortality.[1–8] We describe a rare complication of extensive supratentorial hemorrhages following posterior fossa surgery; review the relevant literature and discus the possible cause of hemorrhage in the present case.

## CASE REPORT

**Figure 1:**
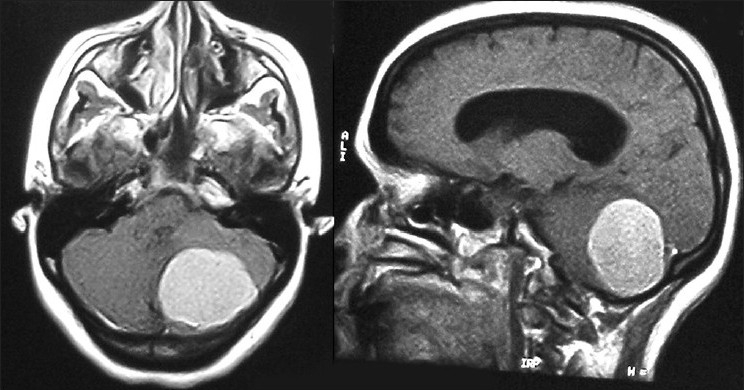
Preoperative MRI brain contrast T1W images showing large posterior fossa tumor on left side with mass effect and obstructive hydrocephalus

**Figure 2:**
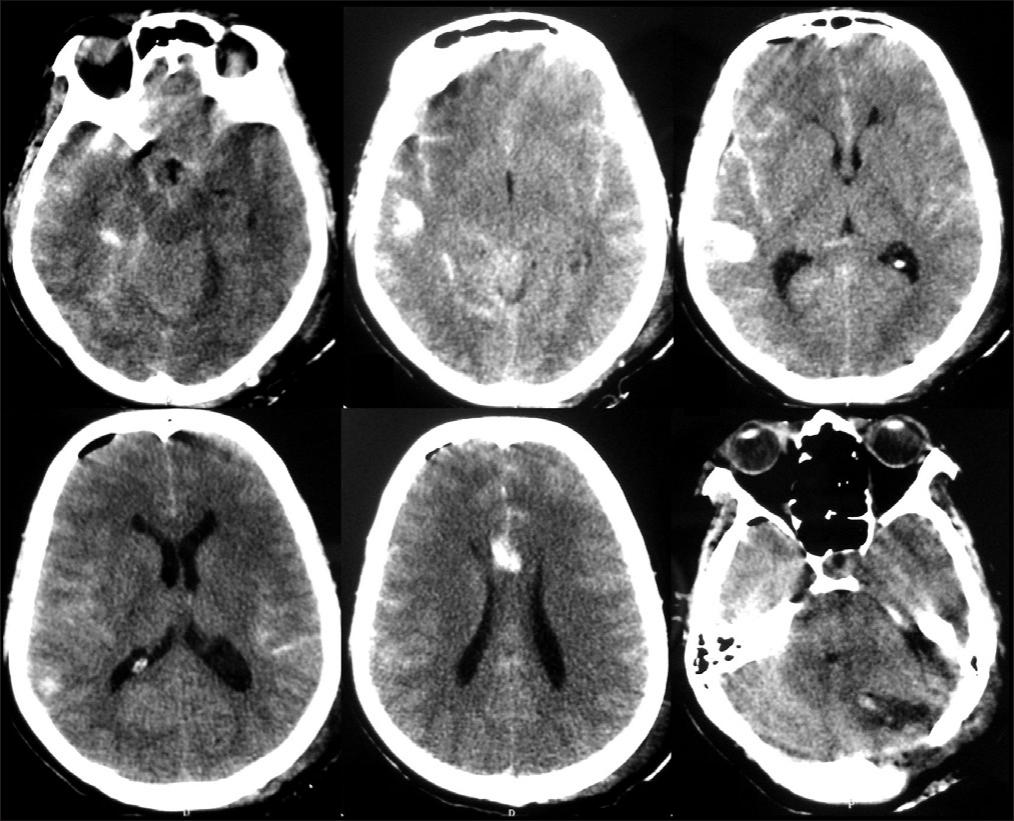
Postoperative CT scan demonstrates extensive subarachnoid hemorrhage with diffuse cerebral edema

A 47-year-old woman presented with headache of 1-year duration that worsened over the last 2 months, progressive ataxia of 2 months’ duration, blurring and diminution of vision of 2 months’ duration and forgetfulness of 2 months’ duration. There was no history of fall, fever or loss of consciousness. There was no history of diabetes, hypertension or suggestive coagulation disorders. Bowel and bladder functions were normal. Her general and systemic examinations were unremarkable. Neurologically she was conscious, alert and oriented to time, place and person. Fundus showed bilateral papilledema, and visual acuity was 6/9 in both eyes. Other cranial nerves were normal. She had left-sided cerebellar signs. There were no focal motor or sensory neurological deficits. Routine blood and biochemical investigations revealed no abnormality. MRI brain with contrast showed a large posterior fossa tumor with obstructive hydrocephalus [Figure 1]. The patient underwent left paramedian suboccipital craniectomy in prone position with left side up. There was a large moderately vascular tumor attached to the dura; the intraoperative course was uneventful, and the tumor was removed totally. In the immediate postoperative period, the patient did not recover from anesthesia and was shifted to the intensive care unit with endotracheal tube. The patient was persistently drowsy even after 3 hours of completion of surgical procedure and had two episodes of generalized tonic-clonic seizure. There was no eye opening, but she could move all four limbs, pupils were bilateral and smaller in size and reacting to light (Glasgow coma scale — eye opening, nil-E1; verbal response on endotracheal tube, V_T_; motor response-localizing to pain, M5). Immediate repeat CT scan showed diffuse subarachnoid hemorrhage spread all over the bilateral cerebral hemispheres with diffuse cerebral edema [Figure 2]. Coagulation profile was normal. The patient was electively ventilated and was started on anti-edema measures (injection mannitol 20%, 100 mL, 6 hourly; injection Furosemide, 20 mg, 12 hourly, continued for 5 days) and anti-epileptics (injection phenytoin, 100 mg, 8 hourly, continued after 5 days as tablet phenytoin). The patient recovered with conservative management without deficits. The patient was found to be doing well at the 6-month follow-up.

## DISCUSSION

Supratentorial hemorrhage following posterior fossa surgery has been reported after surgery for different tumor types, including surgery for meningioma,[5] acoustic tumor surgery,[5] brainstem tumor surgery,[5] fourth-ventricular plexus papilloma surgery,[8] and following posterior fossa surgery for a vermian medulloblastoma.[7] Hemorrhage following posterior fossa surgery can be intraparenchymal[2,7] or extradural,[7,8] or the hematomas can be in the subcortical white matter.[4] Hemorrhage at remote sites after posterior fossa surgery represents a small percentage of hemorrhages, whose mechanism remains largely unclear.[3,9] Changes in intracranial dynamics in the sitting position with disruption of subcortical veins,[4,5,7,9] the possibility of occlusion of carotid or vertebral vessels in the neck by improper positioning of the head leading to intraoperative infarction and to hemorrhage within the infarcted brain after repositioning of the patient,[3,4,7] rapid tapering of cerebrospinal fluid (CSF) pressure after longstanding hydrocephalus[8,10] and clotting disorders could be implicated as causative factors.[5,6,8,11] The clinical manifestation of this complication ranges from subtle neurological deficits to signs of tentorial herniation,[8] and it has been suggested that for any patient who has declining level of consciousness after posterior fossa surgery, supratentorial intracerebral hemorrhage must be included as a differential diagnosis, and computerized tomography should be performed to establish the diagnosis.[2,5,8] The treatment of the supratentorial hemorrhage depends on the type, size, neurological status and location of the hematoma; and depending on the circumstances, the patient will need conservative management or surgical evacuation.[5] Where a coagulation abnormality is suspected, it should be corrected.[6]

To summarize, it needs to be stressed that early postoperative CT scan, optimal management of ventricular pressure and detection as well as prevention of coagulation abnormalities will possibly prevent this life-threatening complication.[8,9]
